# Track density imaging of hypertrophic olivary degeneration from multiple sclerosis plaque

**DOI:** 10.1259/bjrcr.20150299

**Published:** 2016-11-02

**Authors:** Michael J Hoch, Sohae Chung, Girish M Fatterpekar, Ilya Kister, Timothy M Shepherd

**Affiliations:** ^1^Department of Neuroradiology, New York University Langone Medical Center, New York, NY, USA; ^2^Department of Radiology, New York University, New York, NY, USA; ^3^Department of Neurology, New York University Langone Medical Center, New York, NY, USA

## Abstract

A 32-year-old female with relapsing–remitting multiple sclerosis (MS) presented with severe new onset ataxia and diplopia. MRI showed a new inflammatory MS lesion that involved the right dorsal pons and extended into the adjacent superior cerebellar peduncle. The patient improved with aggressive immunotherapy; however, repeat MRI 3 months later revealed a new non-enhancing lesion in the left inferior medullary olive. The differential diagnosis for this new lesion included an MS lesion *vs* hypertrophic olivary degeneration, with infarct or neoplasm as the less likely considerations. We used track density imaging, which provides unprecedented anatomic details based on probabilistic tractography streamlines, to demonstrate apparent changes in the integrity of the dentato–rubro–olivary pathway (Guillain–Mollaret triangle) that were consistent with the diagnosis of hypertrophic olivary degeneration from the antecedent MS lesion involving the right superior cerebellar peduncle. Further medical therapy was avoided, and follow-up MRI 1 year later showed interval involution of the left olivary lesion. This case demonstrates the potential clinical utility of using track density imaging to detect lesion-induced alterations in brainstem connectivity and characterize neurodegeneration in patients.

## Clinical presentation

**Figure 1.**
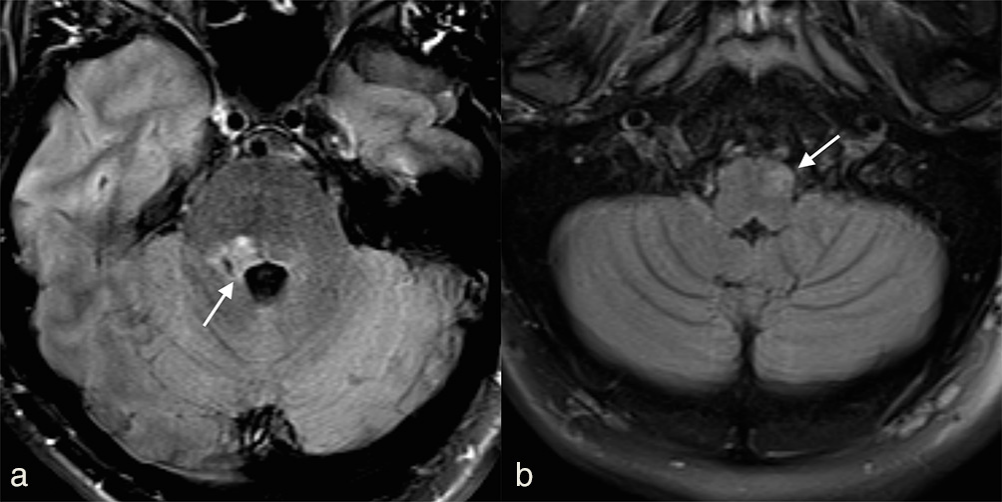
(a) Axial two-dimensional fluid-attenuated inversion-recovery image shows a new demyelinating lesion in the right superior cerebellar peduncle (arrow). (b) Follow-up axial two-dimensional fluid-attenuated inversion-recovery image 3 months later demonstrated new hyperintensity and subtle enlargement of the left inferior olivary nucleus (arrow).

A 32-year-old female with relapsing–remitting multiple sclerosis (MS) presented with severe ataxia and diplopia requiring prolonged hospitalization. Examination at the time demonstrated rotary nystagmus when she was looking up and to the right, incomplete abduction of the right eye, decreased sensation on the right side of the face , left hemibody deficits and severe truncal ataxia (unable to stand without bilateral assistance). MRI of the head showed a new *T*_2_/fluid-attenuated inversion-recovery (FLAIR) hyperintense lesion in the right superior cerebellar peduncle (SCP) (Figure 1a). Aggressive medical treatment with intravenous solumedrol, plasmapheresis and mitoxantrone improved her symptoms. Follow-up MRI performed 3 months later, however, revealed a new *T*_2_/FLAIR hyperintense expansile lesion in the left inferior medullary olive and persistence of the initial right SCP *T*_2_/FLAIR hyperintense lesion (Figure 1b). At that time, the patient had no new clinical symptoms or signs, such as palatal clonus, that correlated to the new brainstem lesion identified on MRI.

## Differential diagnosis

The new expansile lesion in the left inferior medullary olive showed *T*_2_/FLAIR hyperintensity—a new inflammatory MS lesion was considered most likely given the patient’s history. However, a new lesion would be atypical following aggressive treatment with medical therapy. Glial neoplasm seemed unlikely within the short 3-month interval and brainstem infarction would be unusual in this young female with no vascular risk factors and no corresponding changes in mean diffusivity. Hypertrophic olivary degeneration (HOD) was also a primary consideration, given the specific anatomic locations of the medullary lesion and the previously documented afferent demyelinating lesion involving the right SCP. The new medullary lesion may be attributed to degeneration of the dentato–rubro–olivary tract of the Guillain–Mollaret triangle secondary to pathologically altered dentato–rubral fibres, but direct demonstration of tract involvement was not possible with conventional MRI protocols. To help address this diagnostic dilemma, we performed a novel track density imaging (TDI).^1^

## Investigations/Imaging findings

The patient underwent a conventional contrast-enhanced MRI of the head using a 20-channel head and neck coil on a 3 T MRI (Skyra, Siemens Healthcare, Erlangen, Germany). In addition, after informed consent was obtained, we used a novel combination of high angular resolution diffusion-weighted (DW) sequence obtained with simultaneous multislice acquisition diffusion MRI^2^ to produce TDI images of the brainstem structures with a clinically feasible scan time at 3 T. The sequence parameters included 3 mm isotropic resolution, matrix = 80 × 80, field of view = 240 × 240 mm^2^, multiband accelerate factor = 2, 256 diffusion directions with *b*-value = 2500 s mm^−2^, eight images with *b *= 0, repitition time/echo time 3300/98 ms, 42 slices, parallel imaging integrated parallel acquisition technique = 2 with generalized autocalibrating partial parallel acquisition reconstruction (total scan time = 15 min). The DW images were corrected for eddy current distortions using Functional Magnetic Resonance Imaging of the Brain's (FMRIB) Toolbox (FMRIB Diffusion Toolbox ; FMRIB Software Library 5.0.6, www.fmrib.ox.ac.uk/fsl/fdt).^3^

The super-resolution TDI and direction-encoded colour (DEC) TDI maps were generated with 0.5 mm isotropic resolution by using MRtrix3 (Brain Research Institute, Melbourne, Australia, https://github.com/MRtrix3/mrtrix3), which combines the constrained spherical deconvolution (CSD) techniques^4^ with whole-brain probabilistic streamline reconstructions. The relevant fibre-tracking parameters were 100 million tracks randomly distributed throughout the whole brain, 1.5 mm step size, maximum curvature per step = 45°, maximum length = 20 mm and minimum length = 15 mm. The conventional colour scheme was used for track orientation in the patient’s brain: blue (cranial–caudal), green (anterior–posterior) and red (left–right). A short-track TDI method was utilized, where the maximum/minimum track length ratio approached 1, to visualize smaller fibre pathways for more accurate region of interest (ROI) placement in quantification.^5^

**Figure 2.**
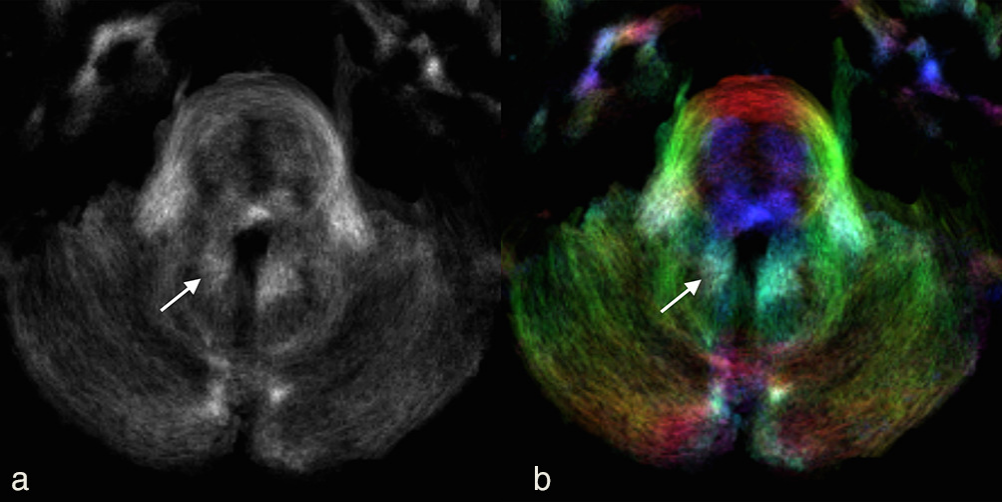
(a) Axial track density imaging and (b) direction-encoded colour-track density imaging at the level of the pons (same level as Figure 1a) 3 months after the initial MRI showing asymmetric probabilistic streamline loss in the right dentate nucleus and denato–rubral track (arrows) compared with the contralateral side.

TDI and DEC-TDI maps demonstrated a visually obvious unilateral loss of track density in the right dentate nucleus and corresponding dentato–rubral tract attributed to a combination of oedema, demyelination and/or axonal loss from the inflammatory MS lesion involving the right SCP (Figure 2). The ROIs were manually drawn and the corresponding track densities per unit volume calculated. The affected right dentato–rubral region had a track density of 11,729 tracks mm^−3^ while the normal contralateral side had a value of 13,037 tracks mm^−3^ (a 10% reduction in probabilistic streamlines).

**Figure 3.**
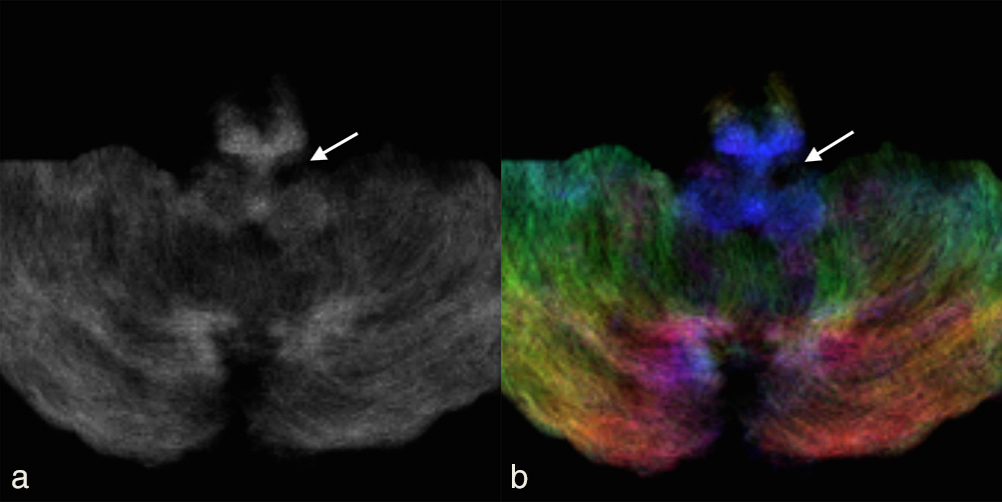
(a) Axial track density imaging and (b) direction-encoded colour-track density imaging at the level of the medulla (same level as Figure 1b) 3 months after the initial MRI showing asymmetric probabilistic streamline loss in the left inferior olivary nucleus and adjacent ipsilateral rubro–olivary tract (arrows) compared with the contralateral side.

The left rubro–olivary region and functionally connected inferior medullary olive, which was pathologically enlarged, also demonstrated unilateral track density loss. Qualitatively, the left inferior olive showed a loss of pixel intensity and blue streamlines on the TDI and DEC-TDI maps (Figure 3). The left inferior medullary olive had a track density of 3329 tracks mm^−3^, while the normal contralateral side had 4410 tracks mm^−3^ (a 25% reduction in probabilistic streamlines). The TDI findings in the corresponding dentato–rubro–olivary pathway, known as the Guillain–Mollaret triangle, supported a final imaging and clinical diagnosis of HOD for the new lesion.

## Treatment

Initial treatment was directed at aborting MS relapse and included i.v. solumedrol courses, plasmapheresis and mitoxantrone chemotherapy. The initial lesion in the SCP resulted in delayed HOD imaging changes that were clinically silent and did not require further therapy upon imaging confirmation of the suspected diagnosis.

## Outcome and follow-up

The patient’s condition slowly improved and she was able to resume full-time employment 3 months after hospital discharge from the initial MS flare. At subsequent follow-up, she remained asymptomatic, except for episodic dizziness. She has been receiving monthly natalizumab infusions for the past 12 months and her MRI of the brain at 1-year follow-up showed the expected evolution of the olivary lesion.

## Discussion

HOD is a unique form of neurodegeneration where loss of afferent input within the dentato–rubro–olivary pathway results in hypertrophy of the inferior medullary olive.^6^ The hypertrophy correlates with an increased number of astrocytes and vacuoles in the affected neurons.^7^ The *T*_2_/FLAIR image hyperintensity within the olive correlates with gliosis. The clinical symptom of classical palatal myoclonus is due to the loss of inhibitory control. The typical imaging findings of HOD usually are present within 6 months following the initial insult. The apparent olivary hypertrophy resolves over 4 years, corresponding with a reduction in the normal number of neurons.^7,8^

The inferior olivary nucleus is situated in the ventrolateral medulla and receives fibres within the central tegmental tract from the ipsilateral red nucleus of the midbrain. The red nucleus receives input from the contralateral dentate nucleus of the cerebellum *via* the dentato–rubral tract within the SCP. To complete the arc, efferent fibres from the olive cross the midline and ascend *via* the inferior cerebellar peduncle into the contralateral cerebellar cortex before projecting on the dentate. This triangle controls fine motor movements and was first described by Guillan and Mollaret^9^ in 1931. Only injury to either one of the two afferent limbs (rubro–olivary or dentato–rubral) results in HOD.

TDI is a novel *in vivo* MRI post-processing technique based on high angular resolution diffusion acquisitions that generate super-resolution images derived by whole-brain probabilistic streamline tractography.^1^ Images of 500 μm isotropic resolution can be created where the pixel intensity reflects the number of probabilistic streamlines traversing the voxel and the colour reflects diffusion streamline orientations per conventional diffusiontensor imaging (DTI). These contrasts have been validated using histology in animal models.^5^

We have combined a track density approach with the recently developed simultaneous multislice acquisition diffusion MRI^2^ to obtain TDI maps of brainstem structures in actual patients using clinically feasible scan times at 3 T. In addition, a sufficient number of DW imaging directions, higher *b*-values and signal-to-noise ratio were achieved to reduce uncertainty in estimating the white matter fibre tracts with whole-brain probabilistic tractography based on the CSD method. It is essential to use an MRI model that accounts for the presence of multiple fibre directions within a voxel, such as the CSD method, to resolve areas of “crossing fibres”.^4^

The super-resolution (0.5 mm isotropic resolution) of TDI provides high-quality fibre pathways with exquisite anatomical contrast. Smaller fibre pathways not directly visualized with conventional DTI, such as those found in the brainstem, are confidently identified. TDI offers the ability to alter the signal-to-noise ratio depending on the chosen sub-voxel resolution and the number of random seed points placed. Even though whole-brain TDI maps are generated from the same DW imaging raw data as routine DTI maps, whole voxel averaging leads to partial volume effects and poor resolution in DTI maps.

TDI can also be used to quantify diseased local structure connectivity so long as diffusion acquisition and post-processing parameters are consistent across patients.^4^ There is debate over which post-processed track density contrast map (short-track, average path length, spherical-deconvolution informed filtering or apparent fibre density) should be used to achieve white matter quantification for larger clinical studies. The short-track map was chosen here owing to its ability to illustrate right *vs* left differences of small structures in a single patient. A manual ROI was drawn around the elongated nature of the dentato–rubral tract and inferior olivary nuclei on axial slices and then compared to the contralateral ROI in the same axial slice. This manual approach helped in confirming tract loss in the expected regions, but it may be better to use automated ROI analysis in future clinical cases or research.

DTI has been used to diagnose the condition of HOD with increased radial and axial diffusivity and decreased fractional anisotropy (FA) in the affected inferior olivary nucleus.^10^ Use of DTI also has been described for demyelination-induced HOD.^11^ To our knowledge, this is the first reported case of TDI being used to support a clinical diagnosis of HOD secondary to a demyelinating lesion. Owing to the super-resolution potential of the TDI methodology, more precise tractography and/or diffusion tract-based contrast of small brainstem structures can be obtained compared with conventional MRI protocols. This case clearly illustrates the potential TDI has for clinical characterization of brainstem pathology and future research investigations of neurodegeneration in the brainstem for other common brainstem pathologies, such as quantitative MRI correlates to parkinsonism.

## Learning points

1. Understanding the underlying mechanisms and confident recognition of HOD on MRI are essential to prevent unnecessary medical interventions.
2. TDI is a novel post-processing super-resolution technique that can generate 500 μm isotropic images based on whole-brain diffusion tractography using clinically feasible simultaneous multislice 3 T MRI diffusion acquisitions.
3. TDI can depict and quantify important complex brainstem anatomy in healthy and diseased individuals, which is not apparent on conventional MRI.

## Consent

Written informed consent was obtained from the patient for publication of this case report, including accompanying images.
